# A Comparison of Census and Cohort Sampling Models for the Longitudinal Collection of User-Reported Data in the Maternity Care Pathway: Mixed Methods Study

**DOI:** 10.2196/25477

**Published:** 2022-03-04

**Authors:** Kendall Jamieson Gilmore, Manila Bonciani, Milena Vainieri

**Affiliations:** 1 Management and Healthcare Laboratory Department of Economics and Management in the era of Data Science, Institute of Management Sant'Anna Scuola Superiore Pisa Italy

**Keywords:** longitudinal studies, mothers, pregnancy, survival analysis, patient-reported outcome measures, patient-reported experience measures, surveys, maternity, postpartum, online, digital health, digital collection

## Abstract

**Background:**

Typical measures of maternity performance remain focused on the technical elements of birth, especially pathological elements, with insufficient measurement of nontechnical measures and those collected pre- and postpartum. New technologies allow for patient-reported outcome measures (PROMs) and patient-reported experience measures (PREMs) to be collected from large samples at multiple time points, which can be considered alongside existing administrative sources; however, such models are not widely implemented or evaluated. Since 2018, a longitudinal, personalized, and integrated user-reported data collection process for the maternal care pathway has been used in Tuscany, Italy. This model has been through two methodological iterations.

**Objective:**

The aim of this study was to compare and contrast two sampling models of longitudinal user-reported data for the maternity care pathway, exploring factors influencing participation, cost, and suitability of the models for different stakeholders.

**Methods:**

Data were collected by two modes: (1) “cohort” recruitment at the birth hospital of a predetermined sample size and (2) continuous, ongoing “census” recruitment of women at the first midwife appointment. Surveys were used to collect experiential and outcome data related to existing services. Women were included who passed 12 months after initial enrollment, meaning that they either received the surveys issued after that interval or dropped out in the intervening period. Data were collected from women in Tuscany, Italy, between September 2018 and July 2020. The total sample included 7784 individuals with 38,656 observations. The two models of longitudinal collection of user-reported data were analyzed using descriptive statistics, survival analysis, cost comparison, and a qualitative review.

**Results:**

Cohort sampling provided lower initial participation than census sampling, although very high subsequent response rates (87%) were obtained 1 year after enrollment. Census sampling had higher initial participation, but greater dropout (up to 45% at 1 year). Both models showed high response rates for online surveys. There were nonproportional dropout hazards over time. There were higher rates of dropout for women with foreign nationality (hazard ratio [HR] 1.88, *P*<.001), and lower rates of dropout for those who had a higher level of education (HR 0.77 and 0.61 for women completing high school and college, respectively; *P*<.001), were employed (HR 0.87, *P*=.01), in a relationship (HR 0.84, *P*=.04), and with previous pregnancies (HR 0.86, *P*=.002). The census model was initially more expensive, albeit with lower repeat costs and could become cheaper if repeated more than six times.

**Conclusions:**

The digital collection of user-reported data enables high response rates to targeted surveys in the maternity care pathway. The point at which pregnant women or mothers are recruited is relevant for response rates and sample bias. The census model of continuous enrollment and real-time data availability offers a wider set of potential benefits, but at an initially higher cost and with the requirement for more substantial data translation and managerial capacity to make use of such data.

## Introduction

Most health care performance data are derived from administrative sources, which can be used to measure the more technical aspects or process measures of maternity care provided to women. However, such data can only capture some dimensions of the quality of care and do not address important features such as patient preferences or overall well-being. These data are also limited since they are collected through interactions with health care providers, do not usually relate to community-based settings, and cannot provide insights outside of formal interactions with a subset of health services, thereby potentially neglecting the contexts in which the real value of care delivered becomes apparent [1-3].

More recently, studies highlighting the importance of priority setting, use of management models, incentives, and other similar efforts have shown that data related to patient-centered measurement may prove to be more useful [4-6]. This includes assessments of patients’ preferences for care, experiences with services, and a range of disease-specific and general health and well-being–related markers. These latter two domains are typically collected through validated tools such as patient-reported experience measures (PREMs) and patient-reported outcome measures (PROMs), respectively [7,8], which can provide responsive and reliable measures of outcomes and experiences [9].

Although they are mainly used in measuring experiences pertaining to or outcomes resulting from acute health care, PREMs and PROMs can be used as longer-term, longitudinal measures. Such measures may include outcomes not tied to specific interactions with health care professionals, such as the case of chronic conditions that are primarily managed by patients themselves. A wide set of characteristics can be measured in this way, including those more relevant to patients’ quality of life than clinical or administrative markers [10]. Technological advances now allow for the systematic collection and analysis of large amounts of such data. Survey data can be collected from large samples at multiple time points; where such models are implemented, the definition of routinely collected data is effectively broadened to include PROMs and PREMs. Although technically feasible, such models have not yet been widely implemented [10].

The maternity care pathway is well-suited to such technology-enabled collection of PROMs and PREMs at scale, since typical performance indicators remain focused on the technical elements of birth, especially pathological elements. However, there is insufficient collection and use of person-centered indicators and of quality measures along the pathway [11], despite evidence that women’s experiences of childbirth go far beyond labor, and that social and psychological aspects of care are important for women [12,13]. Indeed, World Health Organization recommendations underline the importance of woman-centered care to optimize the experience of pregnancy, labor, and childbirth for women and babies through a holistic, human rights–based approach, promoting continuity of care along the pathway [14,15]. Existing efforts to address this information gap include birth cohort studies using online surveys advertised to women through posters and leaflets [16], online surveys advertised through social media [12], and cross-sectional national postal surveys of randomly selected women [17].

For several years, the Tuscan maternal care pathway performance evaluation has adopted a pathway perspective, framed around the person, with inclusion of PREMs collected through periodic patient surveys [18]. More recently, the model was developed through use of digital technology to include both PREMs and PROMs collected at multiple points in time. This model has been implemented in two methodological versions: a cohort-sampling model and a census-sampling model. Both are fully digital, with pregnant women or new mothers enrolled by health professionals and subsequently contacted by SMS text message or email containing survey links.

Previous evaluations of maternity survey data–collection models focused on comparing alternative models based around cross-sectional postal surveys [17]. This study provides insight into two models of longitudinal, personalized, and integrated data collection, the nature of which enable use of analytical methods that have not, to our knowledge, previously been applied in this context. Comparing the performance of the models is useful from several aspects. First, this serves to describe and evaluate each model in isolation, as both offer new features and insights compared to typical maternity data. Second, there remain notable differences in the capabilities and costs between the models.

Thus, the primary research question for this study was: what are the conditions in which a census model is preferable to a cohort-sampling model of longitudinal data collection in the maternity pathway? To answer to this question, we explored (1) whether there are differences in survey participation rates between the two data-collection models; (2) which characteristics of the samples affect participation in the survey; (3) the costs of census and cohort sampling methods; and (4) the strengths and weakness of census and cohort sampling methods, considering the usefulness of the two data collection models to different stakeholders.

## Methods

### Study Design

We used a mixed methods approach to compare several dimensions of census and cohort sampling models of longitudinal data collection along the maternity pathway in Tuscany, Italy. For the quantitative component of the research, we applied survival analysis of survey data, along with identification and comparison of costs, whereas for the qualitative component, we compared the two models with respect to different audiences and purposes.

### Data Source: Longitudinal Data Collection Models

Longitudinal user-reported data collection was carried out from September 2018 to March 2019, using a cohort sampling model. In this model, a predetermined number of women are recruited at maternity hospitals after birth. The sample size was calculated to be representative at the hospital and district levels, considering a 95% confidence level with a 7%-9% 95% CI and 20% follow-up loss. This resulted in a required sample of 3672 women (which was lower than the number ultimately recruited). Exclusion criteria were being a resident outside of Tuscany, preterm delivery (<37 weeks of gestation) or low birth weight (<2500 grams) newborn, or hospitalized in neonatal intensive care. Enrollment was led by midwives in birth hospitals, who were asked to invite every woman (without exclusion criteria) that they supported in birth until the target was reached, after which they were advised to stop. All 24 birth hospitals in Tuscany participated in the recruitment. Each woman was asked to complete six surveys, covering the period from delivery to 12 months later. These surveys were completed after discharge and at 1, 3, 5, 6, and 12 months after childbirth. The follow-up was therefore complete in March 2020.

The second model of longitudinal data collection, which has been in continuous operation since March 2019, uses a census sampling approach. All pregnant women residing in Tuscany who withdrew their pregnancy booklet from a family care center were eligible, with women recruited continuously in these facilities. All 117 family care centers, as the first contact point in the maternal care pathway, participated in the data collection. Midwives enroll all consenting women on an ongoing basis, utilizing the integration between the survey system and the regional information system recording data for pregnancy booklet delivery. Each woman is asked to respond to eight surveys, with data collected at the following points: receipt of the pregnancy booklet; in the second trimester of pregnancy; third trimester of pregnancy; at expected childbirth; and at 1, 3, 6, and 12 months after childbirth. As of the end of 2019, 10,821 women were involved in the survey and answered the first questionnaire.

All surveys are available in Italian and in the seven most commonly spoken foreign languages in Tuscany: English, French, Spanish, Albanian, Arabic, Romanian, and Chinese.

**Figure 1:**
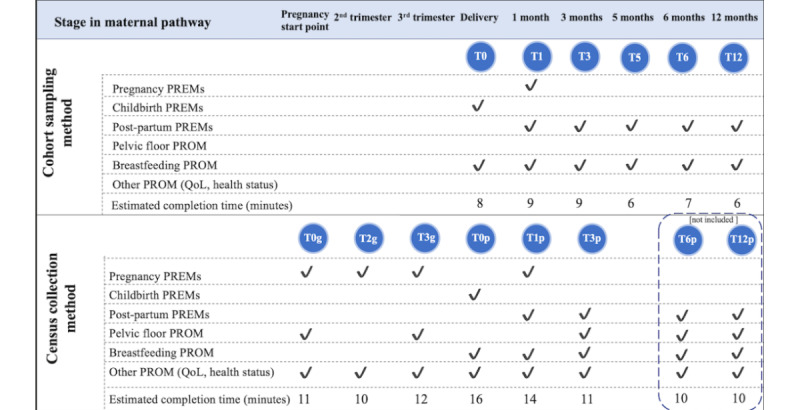
Characteristics of the census and cohort sampling methods. The survey abbreviations in the cohort sampling model refer to the month at which the survey was issued postbirth (ie, T3 in the cohort model was issued at 3 months postbirth). The time points for the census model represent the trimesters in pregnancy (gravidanza in Italian, "g") and months postpartum ("p"). The pregnancy time points represent the trimesters (ie, T3g is the third trimester) and postbirth points represent months after birth, as in the cohort model (ie, T6p is issued 6 months postbirth). The estimated completion time for each questionnaire is based on the upper and lower limits of items (depending on responses to screener questions) and the type of questions per survey. PREM: patient-reported experience measure; PROM: patient-reported outcome measure; QoL: quality of life.

Both models are underpinned by a digitized process. First, the approach is explained to expectant or new mothers, including information about privacy and data uses. Women who consent to join are enrolled in the system, after which surveys are automatically administered according to the stage of the maternity pathway. The questionnaires are personalized according to the gestational period of pregnancy or age of the newborn (only the latter for the cohort sampling model) and include both PROM and PREM items. Each survey addresses topics that are important for pregnant women and new mothers (Figure 1). Unique links to the online surveys are shared by email or SMS, to be filled in by women in a time and place of their choosing using any web-enabled device. Up to three reminders are sent for each survey. Responses are automatically collated and hosted in a secure web platform. Results from the cohort model are presented in a research report at the end of the follow-up period, whereas the census model uses real-time return of data to managers and professionals through a web platform.

For this study, we included data for the cohort model from all mothers recruited between September 2018 and March 2019 (3849 women completed the first questionnaire). All women could have reached the final survey 12 months after enrollment (although some may have dropped out earlier).

For the census sampling model, the data extracted also covered women who joined the data collection period sufficiently long ago to have been able to reach the survey issued 12 months after joining the data collection (wave 6, T3p in Figure 1). The information of women who joined the census sampling model less than 12 months before data collection were extracted and excluded from the analysis. The resultant time period of data collection of the six waves from the census model was from March 2019 to July 2020, with 3935 women included in the study. Data were combined into a single pooled database.

### Data Analysis

Response and attrition rates within each sample population were calculated with reference to the total number of women enrolled from delivery, in the case of the cohort sampling model, and from the first midwife appointment (delivery of the pregnancy booklet), in the case of the census sampling model. The total period of recruitment and data collection for the cohort model was included. Enrollment rates were calculated for both models, using the numbers of enrolled, responding, and total eligible women.

Our methods for interrogating survey attrition rates for women who elected to join the data collection model were based on the framework proposed by Hochheimer et al [19] for evaluating attrition in web-administered surveys. This includes several steps: visualizing attrition using bar charts and survival-type curves; investigation using generalized linear mixed models (GLMMs); and further analysis using survival analysis such as Kaplan-Meier curves, log-rank test, and Cox proportional hazards. Each step provides an additional level of granularity, which can be tailored as appropriate to the study circumstance [19]. We followed all steps except for the GLMM investigating sequential questions, since we were interested in comparing the overall survival in the two models and the factors that are explanatory of survival, rather than determining the significance of changes between sequential questions (in this case, survey waves).

Survival analysis was performed in line with the methodology described above along with processes that were additionally considered appropriate (rather than discrete-time modeling approaches) for our data due to the unequally spaced survey waves [20]. Survival was defined as completion of all eligible survey waves. We created a new entry in the pooled database indicating the failure point for individuals dropping out of the data collection before completion. In this way, the failure event was defined as the occasion in which an individual *could have* responded to a survey *but did not* (rather than the last survey to which they *did* respond). Respondents who completed all eligible survey waves were considered censored. Women in the census model who had an abortion were excluded from analyses. The total population for survival analysis, comprising women from both data collection models, was 7784 individuals with 38,656 observations.

The regression model was built through sequential univariate testing of variables and testing for interaction terms, followed by testing the resulting full model. For categorical variables, the log-rank test of equality was used, with Cox proportional hazard regression used for age (the only continuous variable). The final multivariate model was built using Cox proportional hazards. Covariates included in the final model were age, foreign status (dummy variable), education level (scored from 1-3, with 1=less than high school, 2=high school diploma, and 3=college graduate), employment status (dummy variable), relationship status (dummy variable), and whether the woman had previously had a child (nulliparous, dummy variable).

### Qualitative Comparison

Comparative analysis of the strengths and challenges of each model was performed, informed by theory and by discussions with project stakeholders to reflect their perspectives of the usefulness of the two longitudinal data collection models. These aspects are summarized according to methodological factors, managerial factors, and evaluation factors.

### Cost Comparison

Comparative cost analysis was performed to illustrate the effect of time and number of cohort samples on relative cost-effectiveness, using available program cost data and estimations based on records. Only cost data were used, with no inclusion of benefits from each model, which were considered too diverse for robust quantification. Cost volume breakeven analysis is a common managerial tool to make comparisons between equipment or program alternatives [21]; using this approach, we compared the alternative models to identify the point at which they are similar in terms of simple costs.

Costs were separated according to fixed costs (the basic investment required to establish and implement data collection) and variable costs (those associated with each new group of women, covering all survey waves they will pass through). In reality, there are no separate groups of women for the census model, since recruitment is continuous; for the purposes of cost comparison, we identified the variable costs based on the number of women included in this study.

For the research team, the costs are incurred through survey development and testing, online survey building, user testing, implementation monitoring, design of the web platform, report building, and coordination. For health professionals, the costs are incurred through providing information to/inviting patients; enrolling patients, including data entry; monitoring results; and training in recruitment. For technology and infrastructure, costs are incurred through application programming interface connection development, maintenance of the survey platform, and maintaining the web platform to present data. Communication costs are incurred through provision of information to women and SMS invitations to women.

Statistical analysis was performed using Stata 15 and financial analysis was performed using Microsoft Excel.

### Ethics Approval

The data collection was carried out within systematic surveys developed to monitor women’s experiences, outcomes, and satisfaction with the Tuscan maternity pathways. As such, informed consent was not required, in line with the 2011 Italian guidelines on processing personal data to perform customer satisfaction surveys in the healthcare sector [22].

## Results

### Quantitative Results

A summary of the demographic characteristics for the two population groups is provided in Multimedia Appendix 1. Table 1 summarizes the main statistics for the survey responses.

As shown in Table 1, in the cohort model, 39% of women participated in the first survey wave, with 34% still participating in the final survey wave. Response rates for this model gradually reduced at each survey wave, reaching 87% after 1 year of enrollment. In the census model, 50% of women initially participated, falling to 23% in the final wave. Response rates reduced to 45% after 1 year in the model.

The participation rate represents the proportion of women completing survey waves out of the total eligible population. The eligible population for the cohort sample model is all women who gave birth in the relevant time period, according to hospital administrative data, irrespective of whether they joined the survey or not. For the census model, the eligible population is all women who received the pregnancy booklet and entered the maternal care pathway, irrespective of whether they joined the survey or not. The response rates in both models indicate the proportion of women responding to a survey who were successfully enrolled in the data collection model, completing the first survey (Table 1).

**Table 1:** Survey response descriptive statistics.

Statistic		Cohort model	Census model
**Participation**			
	Total eligible women, N	9827	7826
	Effective participation rate for first wave, n (%)	3849 (39.17)	3935 (50.28)
	Effective participation rate for last survey wave, n (%)	3346 (34.05)	1788 (22.85)
**Response rate, n (%)^a^**			
	T0/T0g	3849 (100.00)	3935 (100.00)
	T1/T2g	3706 (96.28)	3038 (77.20)
	T3/T3g	3633 (94.39)	2463 (62.59)
	T5/T0p	3500 (90.93)	2325 (59.09)
	T6/T1p	3477 (90.34)	1807 (45.92)
	T12/T3p	3346 (86.93)	1788 (45.44)

^a^For the cohort model T0-T12 represent the time from delivery (0) and the months (1, 5, 6, and 12) postbirth. For the census model, T0g, T2g, and T3g represent the month (0, 2, and 3, respectively) of gestation, and T0p, T1p, and T3p represent the month (0, 1, and 3, respectively) postpartum. Also see Figure 1.

### Regression Analysis of Survival Function

Univariate testing of variables indicated that all variables were relevant for further evaluation. No interaction terms were significant.

Testing the assumption of proportional hazards indicated that the impact of the data collection model was not proportional; the final regression model was thus stratified according to the data collection model. All other covariates followed the assumption of proportional hazards.

### Stratification by Survey Type

As indicated in Table 2, all variables except having foreign citizenship were negatively associated with a failure event (not completing all survey waves). Increasing age showed a small reduction in the hazard ratio (HR) per year. Each education level had a reduction in hazard compared to the lowest level. Being employed compared with not employed and being in a relationship compared with being single were associated with a lower propensity to drop out. Women who previously had a child had a lower HR than women in their first pregnancy. It was not possible or appropriate to obtain a single value for the HR survey type on survival, since this changes over time.

**Figure 2:**
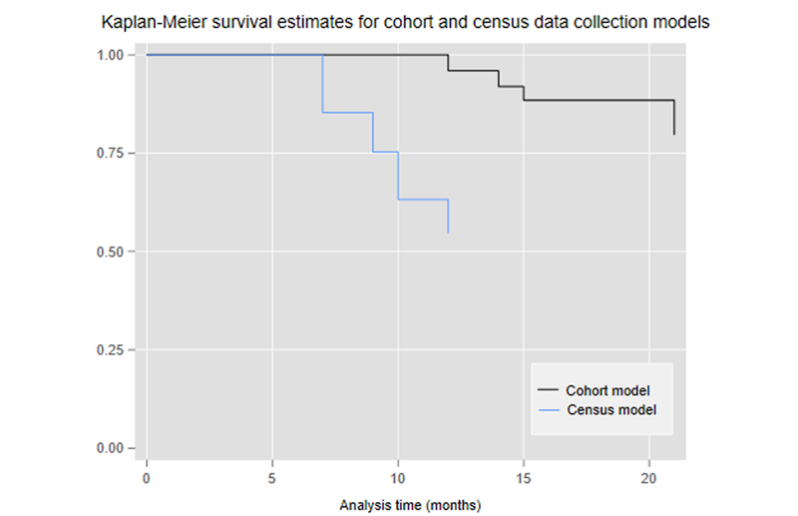
Kaplan-Meier curves of the two survey models.

As shown in the Kaplan-Meier plots for the two models (Figure 2), plots of the survival functions (not shown), and the descriptive statistics, the two data collection models showed different reductions in responses over time. The census collection model showed a large early drop in responses, followed by a less steep, but broadly steady, reduction over time, whereas the cohort model showed small, steady early reductions in responses, followed by a period of very limited reduction.

**Table 2:** Cox proportional hazards and multivariate hazard ratios.

Variable		Hazard ratio (SE)	95% CI	*P* value
Age		0.98 (0.00)	0.98-0.99	.001
Foreign		1.88 (0.11)	1.67-2.12	<.001
**Education level**				
	High school diploma	0.77 (0.05)	0.68-0.88	<.001
	Graduate	0.61 (0.04)	0.53-0.70	<.001
Employed		0.87 (0.05)	0.79-0.97	.01
In a relationship		0.84 (0.07)	0.70-0.99	.04
Nulliparous		0.86 (0.04)	0.79-0.95	.002

### Qualitative Results

There were some common features of the models found, arising from the shared digital administration. These models can be used to collect and manage large volumes of patient-reported data. Such models also enable high response rates, as illustrated in the quantitative results. Additionally, both models are characterized by a comparatively high initial investment followed by low ongoing costs. Both models require analytical resources to derive insight from the data produced.

Digital administration also enables targeted surveys to be shared with expectant or new mothers according to their stage in the pathway, and need not be delivered alongside a specific intervention with a health care professional. This enables surveys to explore, in a timely manner, the aspects of experience or outcomes that are most relevant to people, rather than those based around institutions. This longitudinal design is uncommon in business-as-usual data collection. Additionally, under these models, both PROMs and PREMs can be collected separately or together, providing a more holistic picture of the dimensions of care than one data source in isolation.

There are also features of the two models that differ depending on the administration method, which are summarized in Table 3, categorized according to the relevance to methodology, management, and evaluation in the maternal pathway.

**Table 3:** Summary of methodological, managerial, and evaluative factors in each survey collection model.

Factors		Cohort model	Census model
**Methodological factors**			
	Sample size	Medium to large sample size, predefined	Large, ever-growing sample size with ongoing recruitment
	Representativeness of population	Based on deliveries in birth hospitals	Based on pregnancy at the district level, able to include women from small areas and those who give birth at home or in other settings
	Survey timeliness	Survey at birth requires recall of experiences and outcomes during pregnancy	All surveys relating to the immediate preceding time period
	Bias	Possible sampling bias: enrollment by health professionals after birth may encourage selection of mothers deemed to have had a more positive birth experience	Potential selection bias, although earlier recruitment of mothers reduces the risk of selection based around those deemed to have had a positive birth experience. Selection at first midwife appointment in pregnancy is blind to later experiences and outcomes
	Collection burden	Need for staff training ahead of samples. Enrollment only needed up to a limited period, but is more time-consuming	Ongoing enrollment with less total time spent per health professional. Training only needed for new staff
	Response rate	The initial effective response rate is high, (although lower than that of the census model), with low attrition	The initial effective response rate is the higher of the two models, although drops faster than that in the cohort model
	PROMs^a^ before/after birth	Pelvic floor PROMs are not included as there is no ability to collect prebirth data	Pelvic floor PROMs are included since baseline data at the beginning of the pregnancy are collected
**Managerial factors**			
	Managerial insight	Data provide a snapshot of performance for a certain period of time, enabling lessons to be learned for the following period	Real-time data at different levels of geography enable targeted attention on areas where services need to work better or be better joined up
	Health professional insights	Data provide a snapshot of performance for a certain period of time, enabling lessons to be learned for the following period	Possibility to provide real-time information to different care professionals about the state of delivery of care in their specific area, including highlighting where there are poor experiences or outcomes that professionals could address promptly through their activities
**Evaluative factors**			
	Evaluation models	Enable multidimensional performance assessment	As in the cohort model, and additionally enable inclusion of patient-reported data alongside administrative measures, with contemporaneous reporting periods for both data sets
	Evaluation periods	Data refer to a specific period of collection	Can be used “live” or at any given point in time for evaluating performance
	Analytical approaches	Volume of data can be predetermined according to analytical requirements. Large data sets are possible, enabling advanced statistical models	Continuous collection enables additional analytical approaches (eg, difference in differences) to measure the impact of operational changes

^a^PROM: patient-reported outcome.

### Cost Comparison

**Figure 3:**
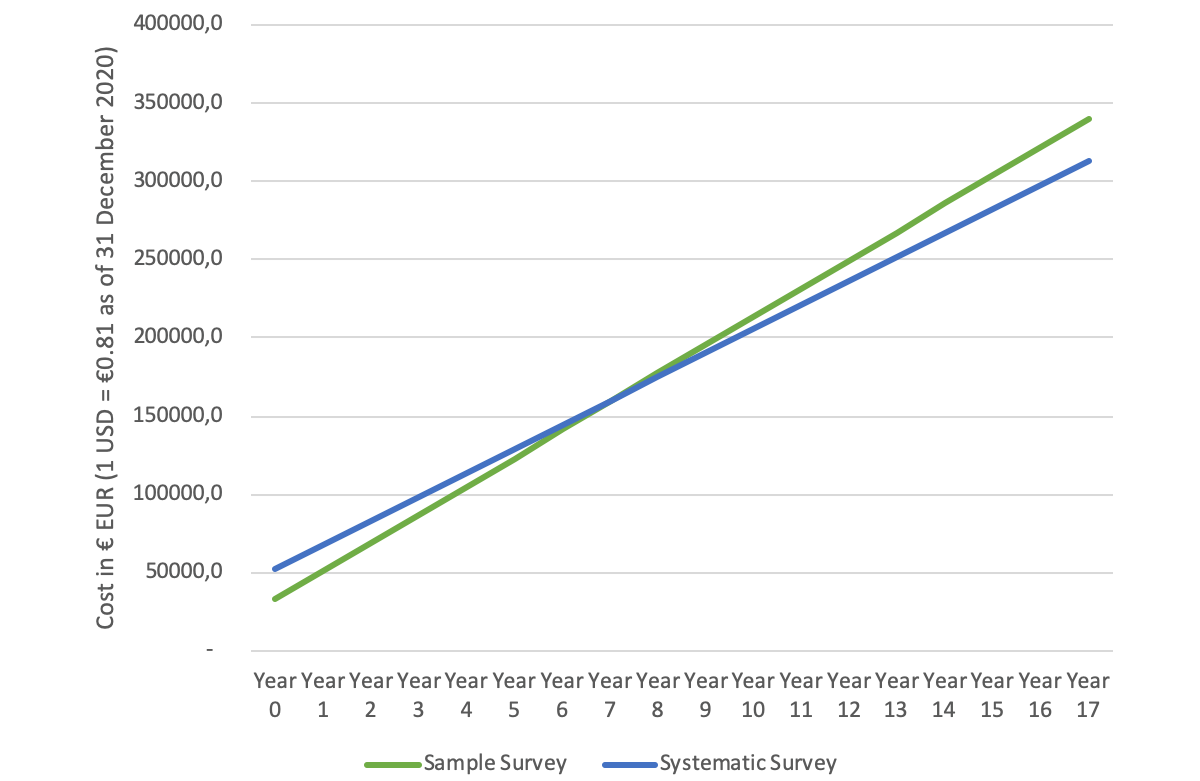
Cost comparison of census and cohort models.

The fixed costs for the cohort and census surveys are calculated at €33,000 and €52,300 (US $1=€0.81 as of December 31, 2020), respectively. Variable costs per group of enrolled women are €18,040 and €15,360. There is therefore a higher initial cost and lower recurrent costs for the census survey compared with those of the cohort model. Projecting costs forward over several years showed that the point at which the census model becomes less costly overall is between years 7 and 8 (Figure 3).

## Discussion

### Principal Results

We use mixed methods to evaluate the performance of two models of data collection for the maternal care pathway, both offering digital longitudinal collection of PROMs and PREMs. The models described, which are fully web-based with longitudinal collection of surveys targeted according to individuals’ positions in the maternal pathway, are interesting from the perspectives of performance management, information, and implementation, and are also of international relevance.

The results of our analyses highlight that both models have some shared benefits. It is established that web-based surveys are cost-effective and provide the same measurement of variables as other collection methods, as well as additional benefits in completeness and data processing. A traditionally observed weakness in web-based surveys compared to postal surveys is their lower response rates (notwithstanding a broader trend of lower study participation across the board) [23-25]. This was not noted in the data collection models described in this study, which showed participation rates at the same level or higher than those of postal collection of maternal patient-reported data, and can be considered to be high in general terms for survey-based research, particularly for online surveys [17,24,26-28]. However, there remained a significant drop-off in responses over multiple survey waves. This could potentially be further improved by shortening the surveys or implementing other adjustments for user experience; further, more targeted feedback should be sought from participating mothers to identify the enablers and barriers to their continued participation in multiple survey waves, particularly in the census model. Since the sample population of mothers is typically fairly young, this model of data collection likely avoids significant exclusion of respondents due to low digital capability as a result of age. However, as shown in the survival analysis, there remain systematic biases with respect to other population characteristics.

The findings from the multivariate regression showed that being less highly educated, not in a relationship, and unemployed were all associated with lower response rates, in line with results obtained over many years in research exploring the impact of participant characteristics on response likelihood [25,29,30]. We also found that having a foreign nationality was associated with a higher dropout risk. Although the data can be risk-adjusted to account for these factors when comparing different reporting areas or periods, it remains an unmet challenge to increase the representation of these groups in patient surveys. There is a risk that maternal services are providing poorer experiences and outcomes or are less responsive to the voices of disadvantaged women in particular, and that this is heightened by lower representation of such women in patient surveys.

The nonproportional hazard functions of the two survey models warrant further investigation to explore the extent to which the changing HRs over time are influenced by the surveys in question (ie, different surveys are differently acceptable and accessible for respondents), stage of pregnancy, and initial recruitment. One potential explanation is a greater selection bias of women in the cohort model (either through unconscious midwife selection or through self-selection, as previously observed in pregnancy cohorts [31]), leading to reduced dropout rates in later stages. Our results suggest that the census collection model provides a more representative sample in the early survey waves, which reduces over time. This is supported by the higher initial participation rate in the census model (49.96%, 3935/7876), but with a lower final effective participation rate (22.70%, 1788/7876) 12 months after enrollment and response rate (45.44%, 1788/3935) after a total of six survey waves. The cohort model had a lower initial participation rate (39.17%, 3849/9827) and a remarkably low attrition rate, leading to an effective participation rate of 34.05% (3346/9827) after six survey waves and a response rate of 86.93% (3346/3849) 12 months after enrollment. The census model surveys are on average longer than the cohort model surveys, which may partially explain the higher attrition rates, although there was no apparent relationship between survey length and attrition within each model. These findings provide lessons for researchers and professionals seeking to collect the views of women in and around childbirth: the timing and mode of recruitment matter. This suggests that studies recruiting women exclusively around childbirth are subject to greater selection bias than those recruiting women earlier in pregnancy. A helpful development in reporting studies using data from new mothers would be to note the effective response rates (as in this study, with reference to the total population of women giving birth), rather than simply those who responded once invited.

The previously observed lower response rates in web surveys are typically based on models that include a postal element and are not digital-only models (ie, individuals are contacted by letter and then provided a web link to respond to the survey). Such mixed models necessarily require an additional step by survey respondents, rather than simply continuing to use the device on which they received the survey link. It is possible that some combination of the simpler, fully digital administration method and the previous in-person enrollment can lead to notably lower attrition rates (eg, 87% response rate at 12 months after enrollment in the cohort model) than have been achieved in other survey models.

From a health system performance intelligence perspective, the overall approach is noteworthy. The longitudinal PROM and PREM data collection in both models is new or uncommon in performance evaluation (typically such data may be collected for specific studies or for limited clinical use). Additionally, the delivery of different PREM and PROM surveys according to patients’ stages in the pathway is a new development in performance measurement, unlike other longitudinal models of PROM collection where the same survey is given at multiple time points. In this way, the information collected is more relevant for assessment and improvement at each point. This could offer new options for using user-reported data in performance improvement, evaluations, and incentive models such as value-based purchasing or as an adjunct to bundled payments, to ensure the patient voice is given appropriate weight [32,33].

### Strengths and Limitations

The application of survival analyses to survey waves is interesting and elucidating. The use of survival analysis to explore attrition in surveys was proposed by Eysenbach [34], as fundamental to growing the “science of attrition,” and has more recently been expounded upon in the context of web surveys [19]. Much of the published literature focuses on the methodology of attrition analyses, with particular attention paid to within-survey attrition [16]. Few studies have used survival methods to explore attrition across multiple survey waves [35] or have described the applied use of such methodologies to inform management practice and implementation. This study focused on the application of survival analysis to real-world survey data collected in multiple waves. This longitudinal experience and outcome data collection are pertinent and useful for measuring performance along a pathway, and can provide insights for managers and clinicians as well as researchers. This analysis thus informs both scholarship and practice in determining the most appropriate data collection models for different purposes.

Limitations of this study include that interpretation of statistical results is not straightforward, partly due to the nonproportional hazard functions of the two models so that a single HR for each model cannot be reported. Additionally, the nature of the multiple-wave survey models means that some women may have missed one or more survey waves without fully dropping out of the data collection. The impact of this is hard to capture. Some women may disengage from maternity pathway services after their initial encounter, resulting in an inaccurate population denominator or less accurate measurement of experiences. Some features of the surveys themselves can also affect response rates, which would require further investigation to distinguish from other model-dependent factors. Tuscan mothers were not included as lay representatives in development of the administration models (although they were involved through their roles as health care professionals and researchers); their involvement could provide further insights into the drivers of attrition (eg, on the impact of survey content).

The two populations of expectant and new mothers are also similar but not identical. These populations were recruited concurrently, not simultaneously, in different settings, and by different individuals (although by midwives in both cases). The demographic table for the two population groups in Multimedia Appendix 1 shows small but statistically significant differences, as would be expected for a large sample size as in this study. In particular, since the two data collection models commence at very different points in the maternal care pathway, women are exposed to different experiences and events at the same time postenrollment (ie, at 9 months), with one group giving birth while the other is caring for a 9-month-old child. This will likely result in different levels of willingness and ability to respond to surveys. Consequently, it is not advisable to make simple comparisons of response rates at the same time point postenrollment; response rates are likely determined by some interacting combination of time since enrollment, period in the maternal care pathway, and demographic characteristics. For example, it is notable that in the survey immediately postpartum, 4% of women in the cohort left the data collection model (1 month after enrollment), whereas 22% of women in the census left the collection (9 months after enrollment). In the first month of the census survey, 23% of women dropped out. There was no 9-month survey in the cohort model, but at 12 months only a further 3% dropped out. These points support the observation that both the timing and mode of recruitment matter. The regression analysis controlled for population differences, but did not consider other factors. As such, in interpreting the results, it is necessary to consider the quantitative data (descriptive statistics, survival analysis, and regression) alongside the commentary about the stages in the pathway addressed by the time points and surveys in the two models.

Regarding the other methodological factors, some results are context-dependent. For example, costs are related to Italy and to the maternity pathway, and variable costs for the census model are imprecise. The qualitative benefits described are derived from the team’s insights and relevant literature rather than from a full stakeholder review; thus, some may have been missed or inaccurately weighted.

### Conclusions

Census collection has a wider set of potential benefits, particularly relating to use of the data as a management tool or for more granular performance evaluation. These benefits are shared by other clinical areas that broadly adopt this model of data collection [10]. However, the cost and effort are higher, which may not be justified in some use cases. A census model also has a higher dropout rate over time, necessitating increased methodological caution in later survey waves. The continuous collection model will be the more cost-effective approach in simple terms if the cohort sample data are collected more than seven times. Although the benefits were not quantified, it is clear that to fully realize the benefits possible from census data, professional and managerial action is required. This requires support including data translation, risk adjustment, and subsequent development of insight. Such efforts have costs. It is therefore probable that census data collection models are more appropriate only in health systems with fairly significant analytical capacity that are able to manage the data and support insight development *on an ongoing basis*. For occasional evaluations or for local areas starting to build their understanding of the reality of experience and outcomes for pregnant women and new mothers, a cohort sampling model may be more cost-effective. In the longer term, two emerging trends in health care will likely shift the balance toward the census model. First, the increasing sophistication of real-time automated analytics and decision-support tools for professionals will reduce the analytical resources required for continuously collected patient-reported data while simultaneously increasing their utility. Second, performance evaluation systems (and reimbursement models) are likely to give greater precedence to measures that matter the most to service users; in such situations, investment in systems akin to the census model will be a priority for all health systems.

## Appendix

Multimedia Appendix 1 Participant characteristics in the cohort and census sampling models.

## Abbreviations

GLMM: generalized linear mixed model
HR: hazard ratio
PREM: patient-reported experience measure
PROM: patient-reported outcome measure
