# Supplementary material for: A Comparison of Census and Cohort Sampling Models for the Longitudinal Collection of User-Reported Data in the Maternity Care Pathway: Mixed Methods Study
Source: JMIR Med Inform. 2022 Mar 4;10(3):e25477. doi: 10.2196/25477 (PMC8933795; doi:10.2196/25477)
Supplement: Multimedia Appendix 1 [file medinform_v10i3e25477_app1.docx]

**Supplementary material -** table of participant characteristics in the cohort and census sampling models.

Percentages are calculated with reference to the total enrolled women for each model, except for the birth type in the census model, which is with reference to the number of women still present in the data collection at that point in time to avoid inaccurate reporting of missing data. Also relating to birth type, in the cohort model this data is reported by midwives at time of enrolment, using hospital administrative codes; in the census model this is self-reported by women who select an option from a list of commonly understood terms.

|  | **Cohort model** |  | **Census model** |  |
| --- | --- | --- | --- | --- |
|  |  |  |  |  |
| **Educational level** |  |  |  |  |
| Low | 528 | 14% | 465 | 13% |
| Medium | 1,690 | 44% | 1,474 | 42% |
| High | 1,631 | 42% | 1,596 | 45% |
|  |  |  |  |  |
|  |  |  |  |  |
| **Employment status** |  |  |  |  |
| Not employed | 1,013 | 26% | 708 | 20% |
| Employed | 2,836 | 74% | 2,825 | 80% |
|  |  |  |  |  |
|  |  |  |  |  |
| **Nationality** |  |  |  |  |
| Italian | 3,400 | 88% | 3,160 | 89% |
| Non-Italian | 449 | 12% | 404 | 11% |
|  |  |  |  |  |
|  |  |  |  |  |
| **Previous birth?** |  |  |  |  |
| Yes | 1,755 | 46% | 1,442 | 41% |
| No | 2,094 | 54% | 2,077 | 59% |
|  |  |  |  |  |
|  |  |  |  |  |
| **Age class** |  |  |  |  |
| <25 | 175 | 5% | 164 | 5% |
| 25-29 | 654 | 17% | 609 | 17% |
| 30-34 | 1,368 | 36% | 1,283 | 36% |
| 35-39 | 1,178 | 31% | 1,089 | 31% |
| >40 | 474 | 12% | 419 | 12% |
|  |  |  |  |  |
|  |  |  |  |  |
| **BMI class** |  |  |  |  |
| Underweight | 297 | 8% | 272 | 8% |
| Normal weight | 2,636 | 68% | 2,444 | 69% |
| Overweight | 664 | 17% | 587 | 16% |
| Obese | 252 | 7% | 261 | 7% |
|  |  |  |  |  |
|  |  |  |  |  |
| **Birth type** |  |  |  |  |
| Spontaneous | 2,768 | 72% | 1,809 | 89% |
| Elective caesarean | 419 | 11% | 19 | 1% |
| Emergency caesarean or caesarean in labour | 316 | 12% | 211 | 10% |
| Forceps or suction | 193 | 5% |  |  |
|  |  |  |  |  |
